# Notes and News

**Published:** 1899-12-02

**Authors:** 


### NOTES AND NEWS.

(Collected and Communicated.)

We understand that about 70 per cent. of the troops who have gone to South Africa have been inoculated against typhoid fever.

The foundation-stone of a cottage hospital has been laid at Wellingborough, which is intended to commemorate the Queen's Jubilee in the neighbourhood.

Recent extensions and improvements have been made at the Radcliffe Infirmary, Oxford. A new operating theatre has been provided, and in the old building better accommodation for the nurses and domestic staff has been arranged. The medical staff has been provided with new quarters, consisting of dining, sitting, and bed rooms. The expenditure in the operating theatre was £2,249, and the other alterations cost upwards of £2,000.

The lamentable deficiency in numbers, so far as regards officers, which has long characterised the Army Medical Service is now beginning to show its true seriousness. With 83 per cent. of its effective strength already abroad, and with another Army Division to mobilise, the question where the medical officers required for the latter purpose are to come from is becoming an anxious matter. Of course, plenty of civilians can be obtained for base hospitals and certain other purposes, and many retired army men are available for examination of recruits. But the whole system of the Army Medical Service hinges on the existence of a proper supply of trained commissioned officers of the Royal Army Medical Corps, and, unfortunately, the supply has run short. For years past the number has been kept so low that the medical officers have not been able to obtain their proper leave, even in times of peace; and now it would seem probable that if there should be anything like a heavy mortality among the medical men in the front, the army will be actually short of officers to fill up the vacancies so caused. The matter is likely to prove distinctly serious.

The Chester Corporation have opened a new isolation hospital at Sealand. The cost of the building is estimated at £19,000.

According to the returns made to the Indian Government the mortality caused by wild animals and poisonous snakes in India continues to be very great, the deaths from this cause in the year 1898 having amounted to 25,166, in addition to which about 80,000 cattle were destroyed by the same means.

The new Victoria wing at the East Suffolk and Ipswich Hospital consists of two new wards, with circular ends to the south, to contain 14 beds in each, with the usual offices. The basement contains men's day-room and servants' hall. The wing cost £6,000, irrespective of furnishing. It was designed by Mr. Bishop, of Ipswich.

We are very glad to find that the sudden and alarming increase in the prevalence of typhoid fever in London which has marked the last four weeks has apparently ceased, the number of notifications having fallen as quickly as they had previously risen, so that they are now once more at an ordinary level for the time of year.

A new infirmary has been opened for Tyrone County, Ireland, at Omagh. The ground floor of the main building is devoted to administration purposes, and contains also two wards for 12 beds. On the first floor are two large, several small wards, and an operating-room. Four more wards are situated in projecting wings. Mortuary and laundry are situated in a detached building. The cost of the building has been £13,000. The architect is Mr. Owen, of Dublin.

The ridiculous attempt of the Leicester Guardians to resist the demands of the Local Government Board and to defy the High Court has come to nothing, and the outcome of the demonstration is that they have at last decided to appoint a vaccination officer. It is to be hoped that now that the power of the central authority has been vindicated the law will be carried out in an orderly fashion. No one can pretend that the present state of the law as to vaccination is satisfactory, but so long as it remains law it ought to be adhered to; and, we may add, submitted to.

The Bethnal Green Board of Guardians have practically completed building an infirmary, which is situated in Cambridge Road, at a cost of £250,000, and the Prince of Wales has been asked to formally open the same. The Guardians have elected the medical superintendent for the institution. There were some 69 applications from all parts of the country. The position carries with it a salary of £500 per annum, house, and lighting. Dr. W. J. Potts, assistant medical officer of Brook Hospital (M.A.B.), Shooter's Hill, S.E., was elected. He is 32 years of age, and is a single man. He holds the following qualifications: M.R.C.S., L.R.C.P., M.D., London; D.P.H., Cambridge. Dr. E. J. F. Moore was also duly elected medical officer for the workhouse.

